# Dose perturbations from implanted helical gold markers in proton therapy of prostate cancer

**DOI:** 10.1120/jacmp.v10i1.2875

**Published:** 2009-01-27

**Authors:** Annelise Giebeler, Jonas Fontenot, Peter Balter, George Ciangaru, Ronald Zhu, Wayne Newhauser

**Affiliations:** ^1^ Department of Radiation Physics The University of Texas M. D. Anderson Cancer Center Houston Texas U.S.A.; ^2^ The University of Texas Graduate School of Biomedical Sciences at Houston Houston Texas U.S.A.

**Keywords:** proton, neutron, fiducial markers, and heavier particle dosimetry

## Abstract

Implanted gold fiducial markers are widely used in radiation therapy to improve targeting accuracy. Recent investigations have revealed that metallic fiducial markers can cause severe perturbations in dose distributions for proton therapy, suggesting smaller markers should be considered. The objective of this study was to estimate the dosimetric impact of small gold markers in patients receiving proton therapy for prostate cancer. Small, medium, and large helical wire markers with lengths of 10 mm and helix diameters of 0.35 mm, 0.75 mm, and 1.15 mm, respectively, were implanted in an anthropomorphic phantom. Radiographic visibility was confirmed using a kilovoltage x‐ray imaging system, and dose perturbations were predicted from Monte Carlo simulations and confirmed by measurements. Monte Carlo simulations indicated that size of dose perturbation depended on marker size, orientation, and distance from the beam's end of range. Specifically, the perturbation of proton dose for the lateral‐opposed‐pair treatment technique was 31% for large markers and 23% for medium markers in a typical oblique orientation. Results for perpendicular and parallel orientations were respectively lower and higher. Consequently, these markers are not well suited for use in patients receiving proton therapy for prostate cancer. Dose perturbation was not observed for the small markers, but these markers were deemed too fragile for transrectal implantation in the prostate.

PACS number: 87.53.Pb

## I. INTRODUCTION

The full potential of external beam radiation therapy can only be realized if the radiation field is accurately targeted on diseased tissue. Poor targeting accuracy may compromise local control of the tumor and increase risk or severity of complications in normal tissues. Many strategies have been proposed to improve targeting accuracy, including the use of kilovoltage images to guide setup and treatment delivery.^(^ ^1^ ^–^ ^4^ ^)^ In many cases there is inadequate soft tissue contrast on megavoltage or kilovoltage images to identify the target tissue. One method to improve targeting is to implant radiopaque fiducial markers in or near the target tissue. Implanted markers have been used extensively in proton therapy^(5)^ ^,^ ^(6)^ and external beam photon therapy.^(^ ^7^ ^–^ ^10^ ^)^

Despite the benefits gained in targeting accuracy by using implanted fiducial markers, recent investigations have revealed that metallic fiducial markers can cause extreme perturbations in the therapeutic proton dose which, in the case of gold fiducial markers, can cause dose to be reduced by as much as 85% from the prescribed amount.^(11)^ ^,^ ^(12)^ A fiducial marker's size, composition, location, and orientation are key factors that should be considered when determining the appropriateness of a marker's use in patients receiving proton therapy. This was illustrated in a recent study by Newhauser et al.^(11)^ in which proton dose perturbations caused by solid cylindrical markers made of gold, stainless steel, and titanium were compared using Monte Carlo simulations. Each marker in the Newhauser study was approximately 1 mm in diameter and 3 mm long, and the study's conclusion was that solid gold markers, while radiographically visible, cast unacceptably large proton dose shadows. Additionally, study results suggested that gold markers with a smaller mass may provide an acceptable solution.

The objective of the current study was to determine the suitability of small helical, gold fiducial markers for use in patients receiving proton therapy for prostate cancer. Specifically, we verified that the markers' visibility on kilovoltage radiographic images was adequate and quantified the proton dose shadows that were cast. We made these determinations by measuring radiographic visibility of the markers using a kilovoltage imaging system and characterizing their dosimetric impact by means of Monte Carlo simulations and film measurements in a phantom.

## II. MATERIALS AND METHODS

### 2.1. Treatment Unit

A commercial proton therapy treatment system (Probeat; Hitachi Limited, Tokyo, Japan) was used for this study. The system included a nozzle to deliver therapeutic beams of high‐energy, passively‐spread proton beams (<250 MeV at nozzle entrance, <32.4cm penetration range in water) and a kilovoltage x‐ray imaging system to radiographically position the patient. Details of the system have been described elsewhere.^(^ ^11^ ^–^ ^14^ ^)^

### 2.2. Fiducial Markers

We tested commercially available helical gold markers (Visicoil; RadioMed Corp., Tyngsboro, MA) of 1 cm in length. Markers with three different coil diameters were considered: small (a 0.04‐mm‐diameter wire, coiled, with inner and outer coil diameters of 0.27 mm and 0.35 mm, respectively), medium (a 0.25‐mm‐diameter wire, coiled, with inner and outer coil diameters of 0.25 mm and 0.75 mm, respectively), and large (a 0.5‐mm‐diameter wire, coiled, with inner and outer coil diameters of 0.15 mm and 1.15 mm, respectively). These markers have been previously investigated for photon beam radiation therapy.^(15)^ To characterize the markers' performance for proton beam radiotherapy, we used established methods, described by Newhauser et al.,^(11)^ ^,^ ^(12)^ with only minor modifications. We simplified our study by focusing on dose shadows in the distal region of the proton dose distribution, since previous studies demonstrated that the largest proton dose shadows occur in this region.

### 2.3. Radiographic Visibility

**Figure 1:**
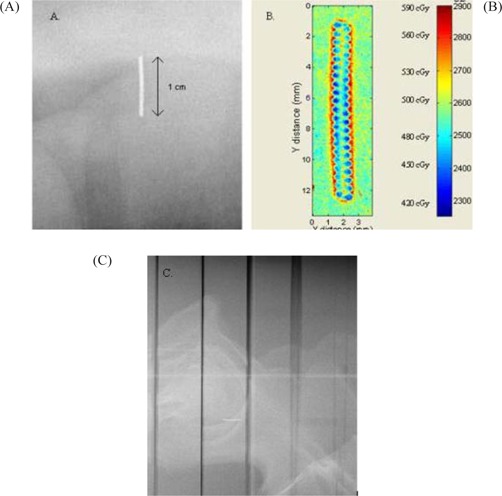
Three representations of a helical gold marker. Kilovoltage radiograph of the medium marker in the presence of bone (anterior) and soft tissue (posterior) in an anthropomorphic phantom. The marker was imaged with the large aperture (18cm×18cm) and the following technique: 110 kVp, 320 mA, 200 ms or 64 mAs. Optical density map with accompanying color scale for the large marker in the distal position. The unperturbed dose was approximately 490 cGy with a standard deviation of film response estimated at 10.474 cGy (i.e. about 2%). The dose behind each coil was approximately 460 cGy, with dose enhancement up to 590 cGy. Kilovoltage radiograph with the medium marker inserted between femoral heads in a RANDO phantom. The marker was imaged with the small aperture (11cm×11cm) and the following technique: 115 kVp, 500 mA, 400 ms or 200 mAs.

To test radiographic visibility, we placed the fiducial markers both on top of (Model 602, 3‐Dimensional Torso Phantom; CIRS, Inc., Norfolk, VA) and inside (Pelvic section, RANDO^®^ Man; The Phantom Laboratory, Salem, NY) anthropomorphic phantoms. Then we imaged them using the kilovoltage patient setup system in the proton treatment room. For imaging on top of the phantoms, each marker was embedded in a thin slab (5mm×75mm×46mm) of extruded polystyrene foam insulation board to provide them with rigid support and a fixed orientation such that their long axes were perpendicular to the axis of the proton beam. This assembly was attached laterally to the spinal region of the thoracic phantom so that when imaged, the marker was imposed on the “spine‐tissue” interface of the phantom (Fig. 1(A). For imaging inside of the pelvis phantom, the markers were rolled in tape and inserted in the ‘Hole Grid’ at a position located midway between the femoral heads. Kilovoltage setup images were acquired using a proton systems patient imaging system (Patient Positioning Image and Analysis System [PIAS]; Hitachi, Ltd., Tokyo, Japan). To approximate a range of prostate treatment conditions, we took lateral images with 18 cm×18 cm and 11 cm×11 cm collimating apertures inserted in the nozzle, and varied the phantom water equivalent thickness (WET) using water equivalent plastic slabs positioned immediately upstream and immediately downstream of the phantom. The additional plastic increased the total phantom WET from 30 cm to 36 cm, increasing the scatter primary ratio (SPR) and decreasing object contrast. In addition, the 18 cm×18 cm aperture size was intentionally larger than the size used for typical prostate treatments,^(11)^ ^,^ ^(16)^ thus further increasing the SPR. The image exposure parameters ranged from 110 kVp, 320 mA, 200 ms, or 64.0 mAs for the thoracic case to 115 kVp, 500 mA, 400 ms or 200 mAs for the pelvic case; typical images for the medium marker can be seen in Fig. 1(A) and 1(C).

### 2.4. Monte Carlo Simulations

Monte Carlo simulations were used to estimate proton dose distributions in the prostate in the presence of fiducial markers using the method described by Newhauser et al.,^(11)^ with only minor modifications (see following description) to accommodate the markers evaluated in this study. A cylindrical shell was used to model the helical markers, using the inner and outer diameters listed in Section 2.2. The uncertainty due to statistical fluctuations in the simulation results was typically 2%‐3% and not more than 5%. To achieve this, $3\times 10^{8}$ proton histories were simulated for each step of the range modulator wheel. Cross‐field dose profiles were generated for the region directly downstream of the fiducial markers; this enabled direct comparison of the simulation results with the corresponding film measurements. The cross‐field dose profiles were tallied in a rectangular mesh of 0.1875mm×0.25mm×0.5mm voxels. Depth dose profiles were tallied in a rectangular mesh of 0.5mm×0.5mm×0.5mm voxels. Dose distributions were simulated and analyzed separately for individual lateral fields and for lateral‐opposed pairs of treatment fields. Irradiation parameters for the simulations closely matched those used in the film measurements described above. In particular, the field size was 14 cm×14 cm, the SOBP width was 10 cm, and the water equivalent penetration range was 29 cm.

### 2.5. Radiochromic Film Measurements of Proton Absorbed‐dose Perturbations

Radiochromic film (Gafchromic EBT ED+, lot #35322–002; International Specialty Products, Wayne, NJ) was used to measure marker‐induced dose perturbations to verify the accuracy of the Monte Carlo simulation model. The markers were placed perpendicular to the axis of the beam on polystyrene foam slabs and exposed at two depths in a solid water phantom. Each depth was irradiated separately. In the first arrangement, polystyrene foam slabs containing the fiducial markers were located 4 cm distal to the center of modulation (COM) of a 10‐cm spread‐out Bragg peak (SOBP) at a water equivalent depth of 27.5 cm. In the second arrangement, the polystyrene foam slabs containing the markers were located 4 cm proximal to the COM of the 10‐cm SOBP at a water equivalent depth of 19.5 cm. In both setups, the film was inserted directly downstream of the fiducial markers and exposed to 500 cGy. The proton field was collimated with a 14.8cm×14.8cm aperture. It had a 10‐cm SOBP width, a range of 28.5 cm WET, and an energy of 250 MeV. In addition, we calibrated film response by irradiating a set of films without markers present. The calibration covered doses from 100 cGy to 500 cGy, which fell within a slightly nonlinear response region of the film, $\text{Dose}(\text{cGy})=3E-05{\text{OD}}^{2}+0.116\text{OD}$, where OD is the optical density times 10000; it was performed at a water equivalent depth of 27.5 cm, with the beam parameters mentioned above.

The film was digitized using a 16‐bit grayscale setting on a photographic scanner (Epson 10000XL; Epson America, Long Beach, CA) with 15.7 pixels per mm sampled. An in‐house software package was used to obtain and analyze these images.^(17)^ The background optical density value was subtracted from each image using an average value taken from a non‐irradiated control film. The resulting optical density files were smoothed by performing a standard box averaging. Perturbation in the proton dose distribution caused by a marker was quantified by subtracting the optical density value in the region of maximum dose perturbation from the optical density value in an unperturbed region of the same film. The unperturbed value was calculated as an average of 3 to 5 random samples in the unperturbed region. The maximum relative uncertainty, or percent standard deviation from the mean, of the film response in the exposed unperturbed region was approximately 2%. Shadow and enhancement regions were defined as regions where perturbations, calculated with respect to the background or unperturbed region of the film, were smaller or greater, respectively, than the background optical density.

## III. RESULTS

### 3.1. Radiographic Visibility

Our tests confirmed radiographic visibility of all fiducial markers evaluated in this study. Typical images can be seen in Fig. 1. Fig.1(A) is indicative of the visibility under conservative conditions due to oversized collimating which resulted in a larger SPR when compared with an image acquired for a typical treatment. The percent contrast for the medium marker in this image is 32% with respect to the mean gray value of the adjacent soft tissue, and 19.5% with respect to the mean gray value of the adjacent bone.

### 3.2. Simulations of Perturbation to Proton Absorbed‐dose Distributions

Monte Carlo simulations provided depth‐dose profiles from a single beam to demonstrate maximum proton dose perturbations. Additional simulations provided data to demonstrate perturbations in dose distribution resulting from a clinically realistic treatment (i.e. radiation delivered using an opposed‐lateral pair of fields, the standard for proton prostate treatments). Perturbations were defined as regions of deviations relative to the unperturbed COM region of the field that were greater than the statistical fluctuations in the simulation.

Dose perturbations from the single‐field simulations are summarized in Table 1. These results qualitatively confirm the previous findings of Newhauser et al.^(11)^: amount of dose shadow depends on marker size, orientation relative to the proton beam axis, and distance from the beam's distal fall‐off location. We also considered the volume of tissue that would be in the underdose region by assuming a rectangular region defined by marker length and width and the measured perturbation for depth. When the markers were perpendicular to the beam axis, we found that the medium marker's maximum shadow was 18%, while the large marker's was 30%. The role that orientation plays in dose perturbation was determined by comparing results for the medium marker at a water equivalent depth of 22 cm; the maximum dose shadows were 18% and 64% for the perpendicular and parallel orientations, respectively. The effect that distance from the beam's distal fall‐off location has on dose perturbation was revealed by comparing results for the medium marker in the perpendicular orientation; the maximum dose shadows were 9% and 18% for depths of 14 cm and 22 cm, respectively.

**Table 1:** Summary of simulated data from single‐field, depth‐dose curves for the large and medium markers.

*Marker Size*	*Orientation*	$Z_{c}(\text{cm})$	$-\Delta D_{\max}(\%)$	$Z_{s}$ (cm)	*V (cc)*
Large	Perpendicular	14	−12.8	1.45	0.083
Large	Perpendicular	22	−30.5	0.90	0.052
Large	Parallel	14	−45.0	1.00	0.058
Large	Parallel	22	−85.3	1.00	0.058
Medium	Perpendicular	14	−9.0	0.75	0.028
Medium	Perpendicular	22	−17.9	0.50	0.019
Medium	Parallel	14	−39.7	0.95	0.036
Medium	Parallel	22	−64.1	0.70	0.026

Markers are oriented parallel or perpendicular to the beam axis at 4 cm downstream of the center of modulation (COM) and 4 cm upstream of the COM. $Z_{c}$ denotes the distance from the phantom edge to the downstream edge of the marker; $-\Delta D_{\text{max}}$ is the maximum dose shadow relative to the unperturbed dose; $Z_{s}$ denotes the distance from the marker's downstream edge to the deepest region of the dose shadow; and *V* denotes the approximate volume of the shadow.

**Figure 2:**
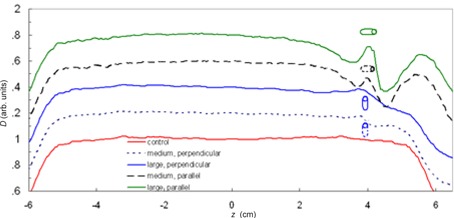
Plot of relative absorbed dose, D, and a function of distance along the beam axes from parallel opposed fields with the large and medium markers. The center of the prostate and the center of modulation (COM) were located at isocenter (z=0cm). The markers were located at z=4cm. For clarity, the curves were offset by adding multiples of 0.2. The dose shadow of each profile is compared to the area at the COM of the control (i.e. the area around z=0cm).

Dose perturbations for the lateral‐opposed‐pair treatment technique are plotted in Fig. 2. These results are for markers located 4 cm from isocenter along the beam axes. The large markers produced perturbations of 44% when oriented parallel to the beam axes and 18% when oriented perpendicularly or in the direction that would have caused the smallest perturbation. Perturbations were not observed for the small markers, but they were judged too fragile for implantation in the prostate and were not considered further. The maximum dose perturbations from the medium markers were 10% and 36% for the perpendicular and parallel orientations, respectively. Because variations in the transrectal implantation procedure and seed migration can lead to marker orientations that deviate from perpendicular, we used the method described by Newhauser et al.^(11)^ and combined results from the two simulated orientations to approximate results for an oblique marker. Using this method, we found that perturbation for the large marker was 31% and for the medium marker was 23%.

### 3.3. Radiochromic Film Measurements of Proton Absorbed‐dose Perturbations

Proton dose perturbations from the large and medium markers were observed distal to the COM. The amount of dose perturbation in this analysis was less because, due to practical experimental reasons, the film was placed directly behind the markers; thus it was at a shallower depth than the point of maximum dose shadow. The resultant perturbation for the large marker was 6.0%; for the medium marker, the measured perturbation was similar, at 5.9%. The uncertainty in these measurements was 2%. In addition to shadowing, the large and medium markers also caused small regions of dose enhancement. These regions of enhancement occurred along the interface of the gold marker and the supporting polystyrene.^(18)^ Fig. 1(B) provides an example of both shadowing behind the marker and dose enhancement in the interface region for the large marker. Percent mean enhancement for the large and medium markers was 3.2% and 2.4%, respectively, with corresponding uncertainties of 4% and 3.5%.

The film measurements and Monte Carlo simulations were in good agreement, which was important because the findings of this study were largely based on results from the simulations. In particular, the images from both methods were qualitatively similar, and the observed dose shadows and enhancements agreed within their respective uncertainties. Simulation results for the region directly behind the large marker included a 5.7% dose shadow and a 2.4% dose enhancement with corresponding uncertainties not exceeding 5%. Thus, agreement between the simulation and film results served to validate the Monte Carlo simulation model and increase confidence in the simulated results presented above. Perturbations less than 2%, our minimum level of detection, were not observed behind the small markers at any depth, nor behind the medium and large markers at the depth proximal to the COM.

## IV. DISCUSSION & CONCLUSIONS

Our results indicate that proton dose perturbations caused by the medium and large helical gold markers exceeded 10%. While the small helical markers did not produce observable dose perturbations, they were deemed too fragile for implantation in the prostate.

Our findings are of clinical significance because they provide cautionary data that encourages the clinician to carefully weigh potential benefits against detriments associated with the use of these markers. In particular, the small dose perturbations observed in this work (<0.1cc) could theoretically compromise treatment efficacy. Our results are similar to those reported by Newhauser et al.^(11)^ ^,^ ^(12)^ for solid gold markers, which also produced unacceptably large dose shadows. Our results suggest that the medium and large helical gold markers should be used with extreme care, if at all, in patients receiving proton therapy for prostate cancer. However, it may be preferable to modify the helical marker to overcome the problems found. Specifically, based on previous work,^(11)^ it appears likely that using a lower density metal in place of gold would preserve acceptable radiographic visibility and mechanical stability while reducing the proton dose shadows to an acceptable level.

One of the possible limitations of this study is the use of a cylindrical shell to model the helical markers in the Monte Carlo simulations. Because of this difference, there is a potential for variation between the simulation and film analysis. However, we believe that this is a reasonable first approximation since the length of gold along the longitudinal axis is large compared to the length of tissue. In both the large and medium markers, the ratio of gold to tissue was conservatively calculated to be 98%. This value was obtained by multiplying the number of turns for a specific marker by the wire diameter and dividing by the marker length. While this could in theory explain some of the difference between measurement and simulation, the difference between the two approaches is more likely due to uncertainty associated with precisely correlating the plane of the film measurements with the corresponding depth in the simulation results.

## ACKNOWLEDEGMENTS

This work was supported in part by Northern Illinois University through a subcontract of Department of Defense contract W81XWH‐08‐1‐0205. The authors would like to acknowledge Richard Wu for his help with the kilovoltage radiographs.
